# Synchronous Two-Stage Hepatectomy With Associated Liver Partition and Portal Vein Ligation in Addition to Low Anterior Resection for Metastatic Rectal Cancer

**DOI:** 10.7759/cureus.21381

**Published:** 2022-01-18

**Authors:** Lisandro Montorfano, Shanna Hutchins, Stephen J Bordes, Conrad H Simpfendorfer, Mayank Roy

**Affiliations:** 1 Surgery, Cleveland Clinic Florida, Weston, USA; 2 Anatomical Sciences, Ross University School of Medicine, Weston, USA; 3 Surgery, Louisiana State University Health Sciences Center, New Orleans, USA; 4 Hepato-Pancreatico-Biliary Surgery, Cleveland Clinic Florida, Weston, USA

**Keywords:** hepatobiliary surgery, low anterior resection, rectal cancer, liver volume, alpps

## Abstract

The associating liver partition and portal vein ligation for a staged hepatectomy (ALPPS) procedure is an excellent treatment strategy for patients with advanced primary or metastatic liver cancer and small liver remnants. This report discusses the surgical management of a 51-year-old male who was diagnosed with stage IV rectal cancer with multiple heterogeneous hypoenhancing solid masses seen in both lobes of the liver consistent with metastatic disease. He completed six cycles of preoperative FOLFOX chemotherapy with Avastin. Follow-up imaging demonstrated a good response. A combined low anterior resection and two-stage hepatectomy with ALPPS were discussed with the patient who agreed to proceed with the plan. He underwent a combined open low anterior resection with colorectal anastomosis in addition to the first stage of ALPPS. The patient tolerated the procedure well, and the immediate postoperative period was uncomplicated. Volumetric assessment of the left hepatic lobe on postoperative day seven demonstrated 26.7% of the original liver volume. The decision was made to take the patient back to the operating room on postoperative day nine for completion of the ALPPS, which entailed a total right hepatectomy. He tolerated the procedure well and was discharged home on postoperative day 16. No complications from the surgical procedure were reported on subsequent follow-up visits.

## Introduction

Colorectal cancer is the third leading cause of death in both men and women in the United States. The disease is expected to cause approximately 52,980 deaths by the end of 2021 [1]. Of those diagnosed with colorectal cancer, roughly half develop hepatic metastases because the portal circulation acts as an effective conduit for metastasis [2]. As such, advances in both colorectal cancer screening, as well as medical and surgical treatment options, continue to be at the forefront of medicine. The associating liver partition and portal vein ligation for staged hepatectomy (ALPPS) procedure is a valid treatment strategy for patients with advanced primary or metastatic liver tumors and small liver remnants [3,4]. This report discusses the case of a 51-year-old male who was diagnosed with stage IV rectal cancer with multiple synchronous heterogeneous hypoenhancing solid masses in both lobes of the liver consistent with metastatic disease. After neoadjuvant chemotherapy, follow-up imaging demonstrated a good response, and the patient was offered surgery. This report details the feasibility and safety of a combined open low anterior resection with colorectal anastomosis and ALPPS.

## Case presentation

**Figure 1:**
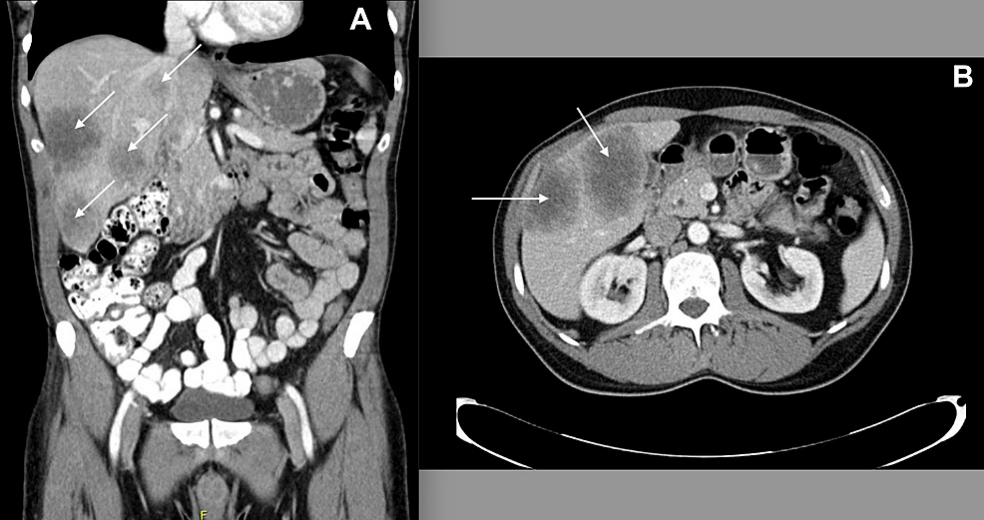
CT imaging of bi-lobar hepatic metastases (white arrows). A: coronal view; B: axial view. CT: computed tomography

A 51-year-old male developed intermittent abdominal bloating with blood in his stool. He underwent a screening colonoscopy which showed a large ulcerated partially obstructing mass in the proximal rectum. The lesion was located at the level of the second rectal valve. Biopsy showed moderate to poorly differentiated invasive adenocarcinoma with N-RAS and APC mutations, stable microsatellite, low tumor mutation burden, K-RAS, and BRAF wild-type. Staging workup showed multiple large synchronous heterogeneous hypoenhancing solid masses in both lobes of the liver consistent with metastatic disease (Figure 1). His functional capacity was classified as excellent (>10 METS), he was well-nourished (albumin 4.0 g/dL), and his liver function was normal. After six cycles of preoperative FOLFOX chemotherapy [FOL: folinic acid, F: fluorouracil (5-FU), OX: oxaliplatin (Eloxatin)] with Avastin, follow-up imaging demonstrated a good response of the primary tumor and multiple liver metastases on both lobes of the liver. The largest mass was located in segment V, which measured 5.2 × 3.3 cm compared to 7.6 × 5.2 cm previously. Volumetric measurements of left lobe liver volumes were performed and reported a left lobe liver volume of 212 cm^2^, which resulted in less than 20% of the total liver volume. The case was discussed on the tumor board, and a combined low anterior resection and two-stage hepatectomy (TSH) with associated liver partitioning and portal vein ligation were recommended to the patient who agreed to proceed with surgery.

**Figure 2:**
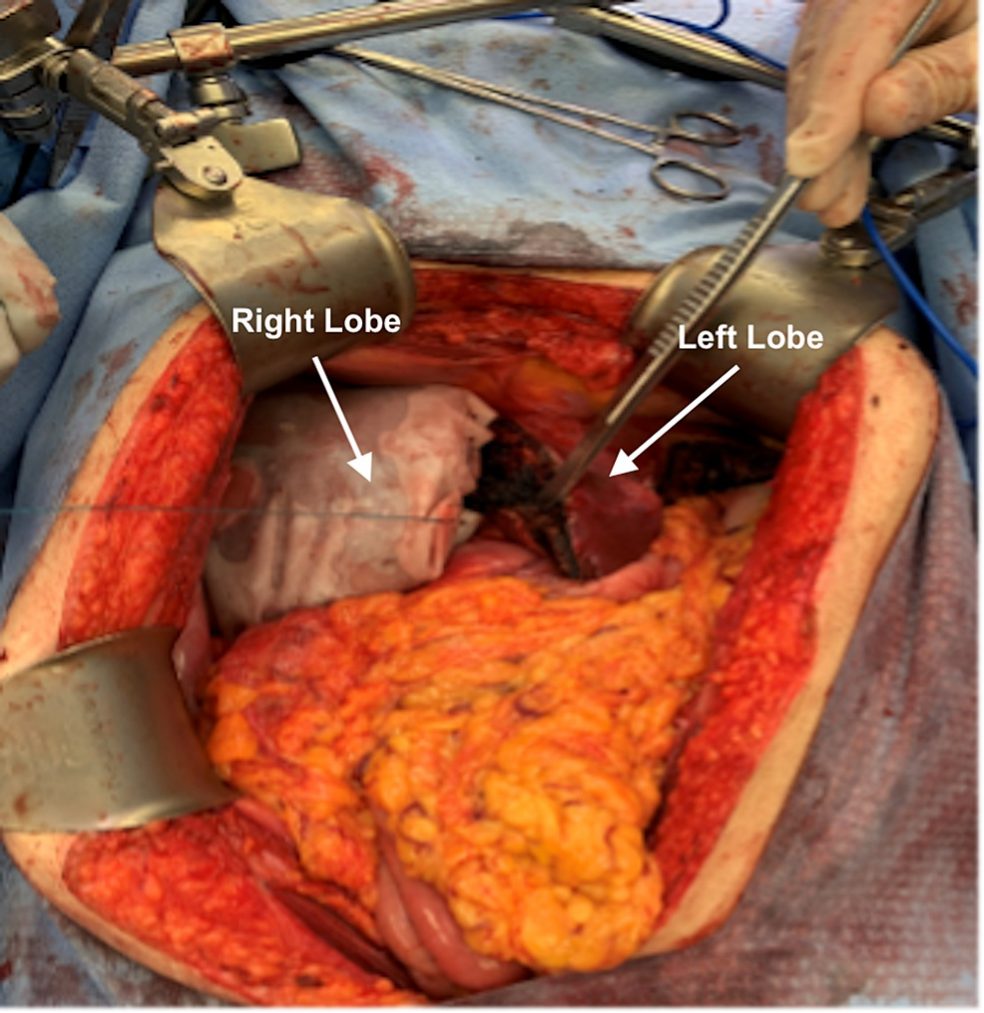
First stage of ALPPS including right portal vein ligation. The right lobe is supplied by the hepatic artery and drained by the right hepatic vein. Rapid hypertrophy of the left hepatic lobe occurs following partition, which directs all portal flow to the left lobe. ALPPS: associating liver partition and portal vein ligation for a staged hepatectomy

A diagnostic laparoscopy demonstrated no evidence of peritoneal carcinomatosis. A midline laparotomy was performed. The liver metastases on segments II and III were identified with intraoperative ultrasound. No additional lesions were seen in the left lateral segment or in segment IV. Multiple large liver metastases were identified on the right lobe of the liver. A standard cholecystectomy was performed. The gastrohepatic ligament was divided, and dissection of the hepatoduodenal ligament was performed. The common hepatic artery as well as the right and left hepatic arteries were identified and isolated using vessel loops. The portal vein was identified. A vessel loop was placed around the right portal vein followed by a bulldog clamp, which demonstrated clear ischemic demarcation along Cantlie’s line. At this point, a partial liver resection of segments II and III was performed. A suprahepatic dissection was carried out. The right hepatic vein, middle hepatic vein, and confluence with the left hepatic vein superiorly were identified. The right lobe of the liver was mobilized from its diaphragmatic and retroperitoneal attachments. The posterior liver was dissected from the vena cava, ligating and dividing the retrohepatic caval tributaries. The liver capsule was scored along Cantlie’s line with Bovie electrocautery. Before performing the parenchymal transection, the colorectal surgery team completed a low anterior resection, and the specimen was sent to pathology. Liver parenchymal transection was subsequently performed. The right portal vein was divided with an endoscopic linear stapler to give time for the contralateral lobe to hypertrophy. The parenchyma was divided along the portal pedicles using a combination of LigaSure device, Aquamantys bipolar sealant, and endovascular staplers (Figure 2). Hemostasis was achieved with Fibrillar and FloSeal. The right hepatic artery was then marked with a looped 0 silk suture for identification during the second and future portion of the ALPPS procedure. At this point, colorectal pathology showed a 3 cm clear distal margin, and a colorectal anastomosis was performed. The patient tolerated the initial procedure well.

**Figure 3:**
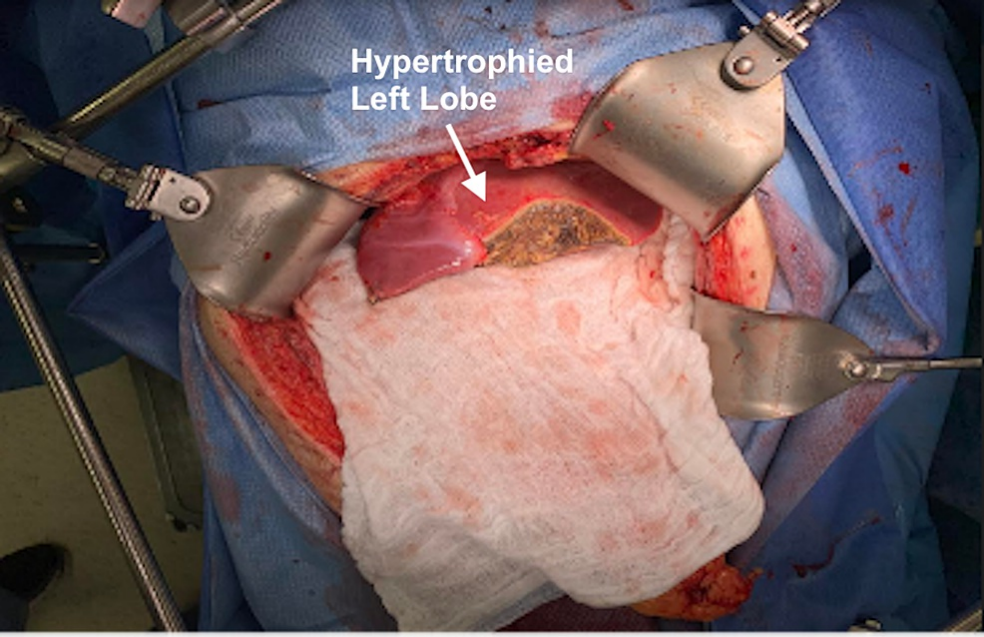
Second stage of ALPPS with a noted hypertrophied left hepatic lobe. ALPPS: associating liver partition and portal vein ligation for a staged hepatectomy

The immediate postoperative period was uncomplicated. Follow-up imaging one week postoperatively demonstrated a partitioned right lobe with compensatory hypertrophy of the left lobe to a size of 369 cm^2^. The patient was taken back to the operating room on postoperative day nine for a total right hepatectomy. The abdominal cavity was entered, and a self-retaining Thompson retractor was placed. There was a small-to-moderate amount of reactive ascitic fluid (most likely related to the previous operation as it was not noted during the index surgery) in the abdomen with no evidence of purulence, bilious staining, or enteric contents in the abdominal cavity. Inspection of the left lobe of the liver demonstrated a well-perfused left hepatic lobe with moderate hypertrophy compared to the initial surgery (Figure 3). Previous resection sites in segments II and III had some fibrinous exudate with no evidence of any bile leak or bleeding. There was no evidence of bile leak at the liver partition between the right and left lobes. The right hepatic lobe was removed. The previously placed silk stitch around the right hepatic artery was identified. This was then doubly ligated and sharply divided. The right hepatic duct was encircled within the liver parenchyma and divided using an endoscopic linear stapler. The remaining parenchyma just above the vena cava was also divided using endoscopic linear staplers. Lastly, the right hepatic vein was divided using an endoscopic linear stapler. Small metastases were noted between the posterior segments VIII and IVA, posterior to the middle hepatic vein. They were excised using sharp dissection and Bovie electrocautery. The patient tolerated the procedure well, and the remaining postoperative course was uneventful. He was discharged home on postoperative day 16. The postoperative period went without complications.

## Discussion

Survival for patients with colorectal cancer heavily depends on the stage at initial diagnosis, with an excellent prognosis for Tumor, Node, Metastasis (TNM) stage I tumors, an intermediate prognosis for operable stages II and III disease, and a poor prognosis for metastatic disease [5]. Treatment involves a combined approach including R0 resection and systemic chemotherapy with FOLFOX and FOLFIRI (leucovorin, fluorouracil, and irinotecan hydrochloride) as the most common regimens [6,7]. For patients diagnosed with operable colorectal liver metastasis (CRLM), surgical resection remains the only potentially curative option and is associated with a five-year survival rate greater than 50% [6,8].

Traditionally, a staged approach has been the preferred treatment method for patients with CRLM [9]. For this approach, either the primary colorectal carcinoma (CRC) (classical approach) or CRLM (reverse approach) is initially resected with or without adjuvant chemotherapy. Later, a subsequent operation is performed for resection of the remaining disease [8,9]. However, with advancements in intraoperative care and surgical technique, the simultaneous approach is gaining popularity. This approach allows for simultaneous removal of the primary CRC as well as CRLM without the need for a second operation [8,9]. Proponents of this approach argue that patients have shorter hospital lengths of stay resulting in decreased procedural cost, as well as similar long-term oncological outcomes when compared to those undergoing a two-stage approach [9]. Others express hesitancy regarding the safety of a simultaneous approach in the setting of a major hepatic or colorectal resection because this can lead to increased morbidity and mortality [9]. In a retrospective study, Tsilmigras et al. suggested that simultaneous resection can be safely performed for select patients undergoing a minor hepatectomy, while this approach should be discouraged in patients requiring major hepatectomy or high-risk colorectal surgery [9].

Regardless of these advances, only 10-25% of patients diagnosed with CRLM will be candidates for surgical intervention [2]. In many patients, CRLM is unresectable due to the extent of disease or inadequate future liver remnant (FLR) [2,4]. Indeed, the function and volume of the FLR is a key indicator of perioperative outcomes, with patients requiring an FLR of at least 20-25% to maintain adequate postoperative liver function [10]. To improve rates of resection in these patients, TSH with or without portal vein embolization and ALPPS procedure were implemented as treatment options [2-4,10]. Of the two procedures, TSH was first performed for patients with bilateral CRLM unfit for resection in a single operation [2]. This involves the resection of tumors in one side of the liver during the first stage, followed by a significant postoperative interval allowing for regeneration of the liver remnant. The remaining tumors are later resected in the contralateral hemi-liver during the second stage [2]. Similarly, the ALPPS procedure is utilized for patients with multi-lobe metastases because it induces rapid hypertrophy of the FLR in one to two weeks [3,4,10]. Consequently, the interval between the first and second operation of ALPPS dramatically decreases as opposed to the TSH interval which requires up to seven weeks between procedures [3,4]. Rapid hypertrophy of the FLR seen with the ALPPS procedure is thought to be a result of the partitioning of the liver parenchyma, which is the key difference between ALPPS and TSH [10]. Multiple studies have focused on comparing the perioperative equivalency of TSH versus ALPPS, although few have focused on long-term follow-up. In a retrospective cohort study, Bednarsch et al. concluded that both ALPPS and TSH exhibited excellent technical feasibility with comparable long-term oncological outcomes [4]. Hasselgren et al. concluded that ALPPS offers improved survival rates compared with TSH, although this significance could be attributed to the higher resection rate achieved with the procedure [3].

Some studies have suggested that ALPPS with partial split can stimulate liver remnant growth similar to complete split with better postoperative safety profiles. However, others have argued that ALPPS can induce more rapid and adequate liver remnant growth with the same postoperative morbidity and mortality rates as partial split of the liver parenchyma in ALPPS (p-ALPPS). A recent systematic review and meta-analysis on ALPPS and p-ALPPS showed that p-ALPPS is safer than ALPPS in patients without cirrhosis and exhibits the same rate of liver remnant hypertrophy. ALPPS appeared to have better outcomes in cirrhotic patients [11,12]. The best technique is yet to be determined.

Regarding minimally invasive surgery (MIS)-ALPPS, the technique has shown good results and can potentially have lower morbidity and mortality when compared to the open technique; however, a limited number of articles can be found on this topic. Reports on MIS-ALPPS have shown high variability in indications and surgical strategies. Patient selection may represent one of the major biases to standardize this practice. Further randomized control studies are needed to better define the patient population that may benefit from this approach [13-15].

Our patient presented with a rectal tumor and bi-lobar liver metastases and subsequently underwent neoadjuvant therapy and low anterior resection with the ALPPS procedure. The postoperative period following each procedure went without complications. The combination of these procedures demonstrated safety and efficacy in the management of patients with colorectal cancer and extensive liver metastases. Further studies are needed to validate our findings. 

## Conclusions

This report details the feasibility of a combined open low anterior resection with colorectal anastomosis and ALPPS, as well as the steps taken to diagnose and treat the patient. ALPPS procedure rapidly increases the FLR, permitting extended hepatectomy for patients with an initially insufficient liver remnant. This procedure was performed at the same time as the low anterior resection without complications. Further studies are needed to confirm our findings.
